# Diarrhoea in the critically ill is common, associated with poor outcome, and rarely due to *Clostridium difficile*

**DOI:** 10.1038/srep24691

**Published:** 2016-04-20

**Authors:** Nikhil Tirlapur, Zudin A. Puthucheary, Jackie A. Cooper, Julie Sanders, Pietro G. Coen, S. Ramani Moonesinghe, A. Peter Wilson, Michael G. Mythen, Hugh E. Montgomery

**Affiliations:** 1 Section of Anaesthetics, Pain Medicine & Intensive Care, Faculty of Medicine, Imperial College London, 369 Fulham Road, London, SW10 9NH, UK; 2 Critical Care, University College London Hospitals NHS Foundation Trust, London, UK; 3 Institute for Sport Exercise and Health, University College London, London, UK; 4 Respiratory and Critical Care Medicine, University Medicine Cluster, National University Health System, Singapore; 5 Centre for Cardiovascular Genetics, Institute of Cardiovascular Science, University College London, London, UK; 6 St Bartholomew’s Hospital, Barts Health NHS Trust, London, UK; 7 Infection Control Office, University College London Hospitals NHS Foundation Trust, London, UK; 8 University College London Hospitals NHS Foundation Trust/University College London National Institute for Health Research Surgical Outcomes Research Centre, London, UK; 9 Microbiology & Virology, University College London Hospitals NHS Foundation Trust, London, UK; 10 University College London Hospitals NHS Foundation Trust/University College London National Institute for Health Research Biomedical Research Centre, London, UK; 11 Critical Care, Whittington Hospital, University College London, London, UK

## Abstract

Diarrhoea is common in Intensive Care Unit (ICU) patients, with a reported prevalence of 15–38%. Many factors may cause diarrhoea, including *Clostridium difficile*, drugs (e.g. laxatives, antibiotics) and enteral feeds. Diarrhoea impacts on patient dignity, increases nursing workload and healthcare costs, and exacerbates morbidity through dermal injury, impaired enteral uptake and subsequent fluid imbalance. We analysed a cohort of 9331 consecutive patients admitted to a mixed general intensive care unit to establish the prevalence of diarrhoea in intensive care unit patients, and its relationship with infective aetiology and clinical outcomes. We provide evidence that diarrhoea is common (12.9% (1207/9331) prevalence) in critically ill patients, independently associated with increased intensive care unit length of stay (mean (standard error) 14.8 (0.26) vs 3.2 (0.09) days, p < 0.001) and mortality (22.0% (265/1207) vs 8.7% (705/8124), p < 0.001; adjusted hazard ratio 1.99 (95% CI 1.70–2.32), p < 0.001) compared to patients without diarrhoea even after adjusting for potential confounding factors, and infrequently caused by infective aetiology (112/1207 (9.2%)) such as *Clostridium difficile* (97/1048 (9.3%) tested) or virological causes (9/172 (5.7%) tested). Our findings suggest non-infective causes of diarrhoea in ICU predominate and pathophysiology of diarrhoea in critically ill patients warrants further investigation.

Diarrhoea is defined as having 3 or more loose or liquid stools per day with stool volume greater than 250 ml/day^1^. In the intensive care unit (ICU) it may result from a range of infective (e.g. *Clostridium difficile* or norovirus), pharmaceutical (e.g. laxatives and enemas) or other non-infective causes (e.g. pancreatic insufficiency and continuous enteral tube-feeding)^1^,^2^. Diarrhoea in the critically ill impacts on patient dignity, increases nursing workload and ICU costs, and exacerbates patient morbidity through dermal injury, impaired enteral uptake and subsequent fluid and electrolyte imbalance^3^,^4^,^5^. Diarrhoea represents grade II (of IV) gastrointestinal (GI) injury according to recent European Society of Intensive Care Medicine (ESICM) guidelines, indicating that digestion and absorption are inadequate to satisfy systemic nutrient and fluid requirements^6^.

Gastrointestinal symptoms are frequent in the ICU^7^,^8^,^9^, and appear associated with impaired outcome in the critically ill^8^,^10^,^11^,^12^. However, few studies have sought to determine the prevalence of diarrhoea in critically ill patients, the likelihood of identifying an infectious origin, or the clinical impact of diarrhoea on ICU length of stay (LOS) or mortality. No previous studies have sought association with antecedent laxative use, which has a potential aetiological role.

We thus aimed to (i) characterise the prevalence of diarrhoea in ICU in a large consecutive patient cohort, (ii) define the proportion in whom an infective agent was identified, (iii) report association with laxative or enema use, and (iv) explore the association of diarrhoea with ICU outcome.

## Results

### Prevalence

**Figure 1:**
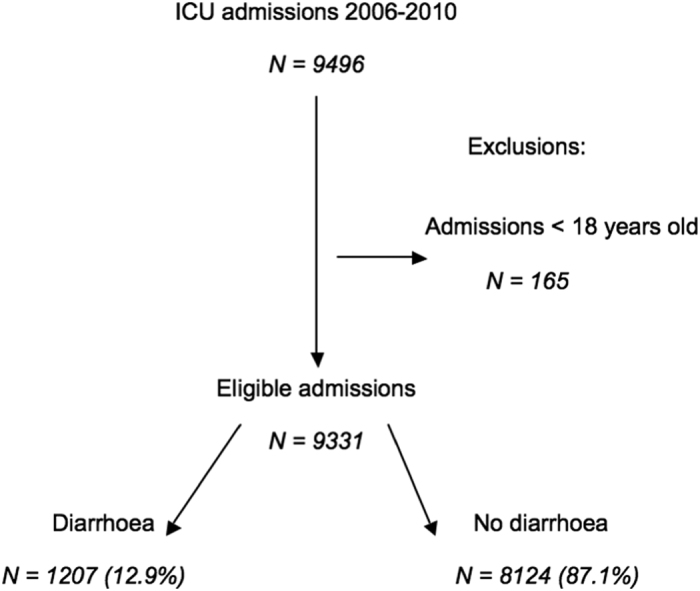
Flow chart of patient enrolment.

Between 01/01/2006 and 31/12/2010, 7697 patients with 9331 admissions meeting the inclusion criteria were admitted to University College Hospital (UCH) ICU (mean age ± standard deviation (SD) 58.6 ± 17.7 years, 55.0% male, 66.9% from surgical admissions, median (interquartile range (IQR)) Acute Physiology and Chronic Health Evaluation (APACHE) II score 16 (11–22)). Diarrhoea affected the patient in 1207 (12.9%) of these admissions (Fig. 1). The demographics for the study group are shown in Table 1. The relationship between diarrhoea and type of surgery (gastrointestinal vs non-gastrointestinal) is shown in Supplementary Table S1. The prevalence of diarrhoea in ICU re-admissions was 23.1% (178/772), compared to 13.3% (85/637) in re-admitted patients on their first admission and 11.9% (946/7922) of patients with only one ICU admission.

### Infective aetiology

Of the 1207 admissions associated with diarrhoea, 111 (9.2%) had pathological microbiological stool samples (‘positive samples’): 6/1149 (0.5%) stool samples sent for microscopy/culture, 97/1048 (9.3%) samples sent for *C. difficile* toxin or antigen, and 9/172 (5.2%) samples sent for virological analysis. Positive stool cultures were for *Salmonella* (4), *Campylobacter* (2) and *Candida albicans* (1). The single case with *Candida albicans* was not utilised in analysis of infective diarrhoea, as this organism was deemed unrelated to diarrhoea pathogenesis. Positive virology samples were all for norovirus (9). All norovirus cases were community or hospital-acquired, and none originated from ICU.

Out of 97 positive *C. difficile* samples, 27 (27.8%) were antigen positive only and 70 (72.2%, involving 67 patients) were toxin positive (Table 2). *C. difficile* was thus the most commonly diagnosed infective cause of diarrhoea, occurring in 97 admissions, involving 94 individual patients (mean age ± SD 65.1 ± 16.2 years, 60.8% male, 53.6% medical admissions, median (IQR) APACHE II score 22 (17–29)). Amongst those affected, 21 were post-operative patients, with the most common medical reasons for admission being respiratory failure (28/97) or sepsis (21/97).

One patient tested positive for both *C. difficile* and norovirus during the same ICU admission. The prevalence of C. difficile (97/9331) and norovirus (9/9331) was 1.0% and 0.1% respectively. The prevalence of *C. difficile* (23/772) and norovirus (1/772) during re-admissions was 3.0% and 0.1% respectively.

### Laxatives, Suppositories and Enemas

Of 9331 ICU patient admissions, 1635 (17.5%) received laxatives during their stay. Some 244/1207 (20.2%) admissions with diarrhoea received laxatives ≤24 hours prior to its onset, while 13/97 (13.4%) admissions with positive *C. difficile* samples were receiving laxatives *within* 24 hours of their positive *C. difficile* sample.

Overall, 1219 (13.1%) of ICU admissions received enemas/suppositories. Of the 1207 diarrhoea cases, 137 (11.4%) received enemas/suppositories ≤12 hours prior to its onset.

### Clinical outcomes

The median (IQR) ICU LOS was 1.9 (0.9–4.5) days and ICU mortality was 10.4% (970/9331). When compared to admissions without diarrhoea, admissions with diarrhoea experienced longer ICU LOS (median (IQR) 9.5 (4.6–20.2) vs 1.7 (0.9–3.2) days, p < 0.001) and greater ICU mortality ((22.0% (265/1207) vs 8.7% (705/8124), p < 0.001).

Median ICU LOS for patients with *C. difficile* was 17.4 (8.4–32.3) days. 26 out of 94 (27.6%) patients with either *C. difficile* antigen or toxin positive samples died during their ICU admission. 23 out of 67 (34.3%) patients with *C. difficile* toxin positive samples died during their ICU admission, which was not significantly greater compared with other admissions of patients suffering diarrhoea during their ICU stay after adjusting for APACHE II score (odds ratio (OR) 1.41, 95% confidence interval (CI) 0.87–2.30, p = 0.168). No patients required surgical colonic resection for *C. difficile* infection.

We performed multivariate regression analysis to adjust for confounding imbalances between diarrhoea and non-diarrhoea groups. Using ordinal logistic regression analysis, the unadjusted odds of a longer ICU stay in patients suffering diarrhoea was 16.3 (95% CI 14.39–18.46, p < 0.001). Increasing APACHE II score was associated with increased ICU stay, while patients admitted to ICU due to operative intervention had significantly shorter ICU LOS compared to all other causes, except for haemorrhage (Table 3). Adjusting for age, referring specialty, APACHE II and reason for ICU admission (including operative intervention to adjust for post-surgical patients with expected short ICU stays), ICU LOS was significantly greater in patients suffering diarrhoea than those without (OR 9.48, 95% CI 8.32–10.81, p < 0.001), the adjusted mean ICU stay estimated to be 11.6 days greater (mean (standard error) 14.8 (0.26) vs 3.2 (0.09) days, p < 0.001).

**Figure 2:**
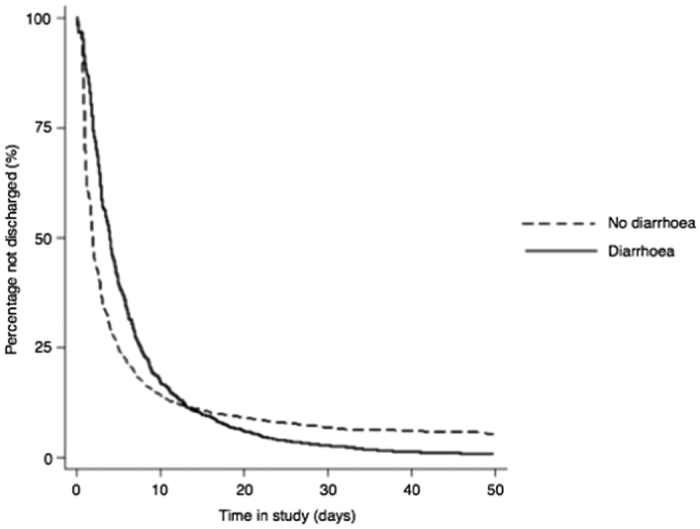
Kaplan-Meier estimate of time to discharge for admissions of patients suffering diarrhoea during their intensive care unit stay vs admissions not suffering diarrhoea using a Cox proportional hazards model with diarrhoea as a time dependent co-variate.

We assessed the relationship between ICU LOS and time from ICU admission to diagnosis of diarrhoea using a Cox proportional hazard model with diarrhoea as a time-dependent covariate (Fig. 2). The proportional hazards assumption did not hold as hazard curves crossed at 13 days. Analysis before and after this cut point showed admissions of patients suffering diarrhoea early in their ICU stay (within 13 days) were less likely to be discharged than admissions not suffering diarrhoea (hazard ratio 0.91, 95% CI 0.83–0.99, p = 0.03), but admissions of patients suffering diarrhoea later in their ICU stay (after 13 days) were more likely to be discharged from ICU than those not suffering diarrhoea (hazard ratio 3.15, 95% CI 2.65–3.74, p < 0.001).

**Figure 3:**
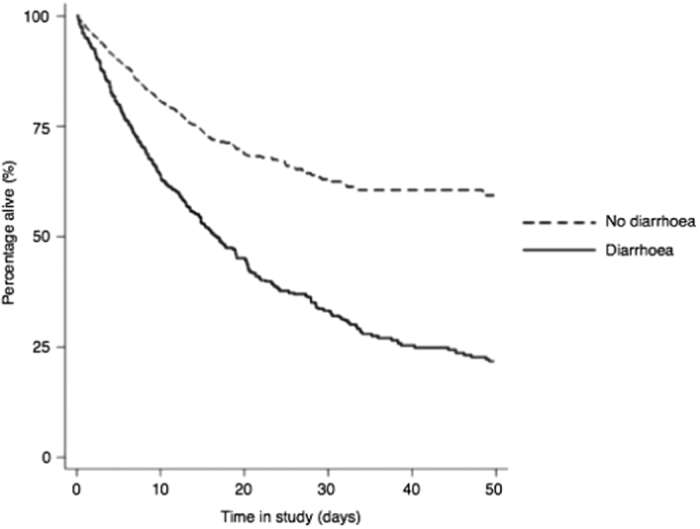
Kaplan-Meier survival curves for admissions of patients suffering diarrhoea during their intensive care unit stay vs admissions not suffering diarrhoea using a Cox proportional hazards model with diarrhoea as a time dependent co-variate.

The relationship between diarrhoea and mortality was analysed by modelling data as time to death using a Cox proportional hazard model with diarrhoea as a time-dependent covariate (Fig. 3). The hazard ratio of mortality for admission with vs without diarrhoea was 1.99 (95% CI 1.70–2.32, p < 0.001). Higher ICU mortality was observed for older patients, those with a medical referral, and increasing APACHE II score. Patients admitted to ICU due to operative intervention had significantly lower ICU mortality compared to all other causes (Table 4).

## Discussion

Our study is the largest yet to describe the prevalence of diarrhoea in the ICU. We found diarrhoea to be common (12.9% prevalence) and to be associated with increased crude ICU LOS and mortality, both persisting after adjustment for severity of illness and other potential confounding factors. A low yield (9.2%) for microbiological/virological stool investigations and low prevalence of *Clostridium difficile* (1.0%) and norovirus (0.1%) suggest non-infective causes may be playing a significant aetiological role. In this regard, over one-fifth of patients received laxatives (20.2%) and/or enemas/suppositories (11.4%) prior to diarrhoea onset.

The high prevalence of diarrhoea (12.9%) in our study of 9331 consecutive ICU admissions is consistent with established literature, which reports a prevalence of 9.7–41%^3^,^7^,^13^,^14^. Indeed, given that our diagnosis of diarrhoea depended upon a stool sample being sent, we may have underestimated its prevalence, especially of milder cases. The baseline demographics for our patient cohort suffering diarrhoea (median [IQR] 62.8 years [49.7–73.2], 56.2% male) were similar to a 10-year retrospective analysis of 5260 ICU patients from 3 ICUs in France (median [IQR] 67 years [56–76], 61.5% male)^14^.

Our low yield (9.2%) of positive stool samples and low yield (9.3%) of positive *C. difficile* samples compares with a yield of 13.5% (69/512) positive *C. difficile* samples from the French ICU patient cohort^14^, which only analysed stool samples 72 hours after ICU admission exclusively looking for *C. difficile* infection. We looked at the index sample sent from patients at any time from ICU admission. Taken together, these findings suggest the role of non-infective causes of diarrhoea warrants further study. While the introduction of *C. difficile* antigen testing for the last 19 months of the study might have increased the total number of *C. difficile* positive cases, the total number (n = 97) and percentage (9.3%) of *C. difficile* positive cases over the study period remains small and in keeping with our overall conclusions. If the *C. difficile* infection rate was based on *C. difficile* toxin-positive cases only (n = 70), the prevalence of *C. difficile* infection in our cohort is even lower (0.8%).

Our study is the largest to look at laxative and enema/suppository use prior to episodes of diarrhoea. Over 20% of patients suffering diarrhoea had received laxatives or enemas/suppositories immediately prior to diarrhoea onset, suggesting a possible aetiological role. There is sparse literature regarding the non-infective aetiology of diarrhoea in critically ill patients. A 1-year prospective study of ICU patients found a 41% incidence of diarrhoea with increased incidence in patients receiving nasogastric feeding and no increased incidence in those receiving antibiotics^3^. A multi-centre observational study in 37 Spanish ICUs on 400 patients admitted to ICU who received enteral nutrition found 14.7% of patients suffered diarrhoea^7^, while a 3-month prospective study of 39 patients receiving enteral nutrition found patients suffered diarrhoea on 38% of feeding days^13^. A 1-month prospective study of ICU patients in Spain found 21.6% of 162 patients suffering diarrhoea received laxatives during their ICU admission^15^. A 2-month prospective study of 278 ICU patients in Switzerland, observing risk factors other than laxatives and *C. difficile* infection in the first 14 days of ICU admission, found enteral nutrition >60% of energy target, use of antibiotics and anti-fungal drugs as independent risk factors for developing diarrhoea in ICU^16^. There was no significant relationship between type of surgery (gastrointestinal vs non-gastrointestinal) and admissions of patients suffering diarrhoea in both the total study population (p = 0.96) and the subgroup of critical care admissions due to operative intervention (p = 0.68, Supplementary Table S1).

Patients suffering diarrhoea had significantly longer ICU LOS and ICU mortality, even after adjusting for confounding factors. Diarrhoea remained independently associated with an 11.6 day increase in ICU LOS (p < 0.001). Few others have sought such association, although *Clostridium difficile* infection has been reported to be associated with an estimated (non-statistically significant) 6.3 day increase in ICU LOS^14^.

The association of diarrhoea with ICU LOS could be explained in one of two ways. Firstly, those staying longer might be at greater risk of developing diarrhoea. Alternatively, the presence of diarrhoea might itself be causally associated with increased LOS. However, our analysis revealed that patients suffering diarrhoea early in their ICU stay (within 13 days) were less likely to be discharged than patients not suffering diarrhoea (hazard ratio 0.91, p = 0.03), but patients suffering diarrhoea later in their ICU stay (after 13 days) were more likely to be discharged from ICU than those not suffering diarrhoea (hazard ratio 3.15, p < 0.001). This suggests time from ICU admission to developing diarrhoea was not a confounding factor. In our institution, persistent diarrhoea is not a common reason for keeping patients in the ICU if the patient is euvolaemic, haemodynamically stable and not requiring aggressive ongoing fluid replacement. Delayed discharge through lack of single room accommodation on the general wards is comparable to other United Kingdom ICUs, with only 25% of general ward beds at UCH having single room accommodation.

The crude mortality rate of patients with diarrhoea was 22%, which is similar to findings from studies observing outcomes from *Clostridium difficile* infection in ICU patients^14^,^17^,^18^,^19^,^20^. The adjusted mortality risk for patients suffering diarrhoea in ICU was 2-fold greater (hazard ratio 1.99, p < 0.001) than patients not suffering diarrhoea at any time point during ICU admission.

We cannot ascribe the increased ICU LOS and ICU mortality with which diarrhoea was associated to this condition itself. However, this association remained after adjusting for confounding factors. Further, there is good rationale for believing that diarrhoea could have such a direct impact. Diarrhoea is shown to impair nutrient intake by the enteral route in ICU patients, exposing patients to undernutrition through enteral feeding intolerance^8^,^12^,^21^. Reduced enteral intake and resulting malnutrition is shown to be an independent risk factor for in-hospital mortality^22^. Further, it causes dermal injury, impaired enteral uptake and subsequent fluid and electrolyte imbalance^3^,^4^,^5^,^6^.

There are several limitations to our retrospective study. Firstly, the prevalence of diarrhoea may have been higher than reported, if diarrhoea occurred but no stool sample was sent. However, with protocols in place, we think such cases are likely to be few. We may have underestimated the number of infective cases given that we only studied the results from the index stool sample, and sequential samples may have yielded positive results. We did not explore all causes of diarrhoea such as the influence of enteral feeding and antibiotics for which data were not fully available from computerised records. Other potential confounding factors, such as patient co-morbidities, levels of organ support and incidence of nosocomial infections, could not be accounted for which may have caused an estimation bias with our results. We recommend a full prospective study to observe the influence of factors such as type of feeding, co-existing infection, and presence of diseases altering stool frequency on the development and duration of diarrhoea in critically ill patients.

Diarrhoea increases ICU workload and healthcare costs, causes patient indignity and morbidity, and may be associated with increased ICU LOS and mortality. ESICM comment that GI tract changes in critically ill patients remain poorly understood, while a lack of markers of GI function has limited progress in exploring GI dysfunction^6^. Studies are clearly needed to increase our knowledge of GI tract pathophysiology which predisposes ICU patients to changes in bowel function. Increased understanding of changes in bacterial flora, GI tract mucosa and GI tract perfusion^23^ may inspire insights into potential biomarkers and novel therapeutic targets, and enable evidence-based guidelines to reduce the clinical and financial burden of diarrhoea on critically ill patients.

Meanwhile, reviewing gastrointestinal function should be a routine part of general housekeeping for intensivists. Early recognition, appropriate investigation and prompt treatment are necessary to reduce the burden of diarrhoea on the already fragile ICU patient. Increasing evidence suggests early protocolised and goal-directed care can improve organ function and patient outcomes during critical illness^6^,^21^,^24^,^25^,^26^. Once diarrhoea is recognised, clearly defined protocols should be implemented which identify the cause, reduce risk of transmission of infectious agents, and treat reversible aetiology. Diarrhoea in the critically ill is likely to be multifactorial. While the importance of excluding infective causes cannot be understated, the low positive yield of stool investigations in our study suggests non-infective causes should also be identified. Rational stool sample testing should be used to balance clinical risk with unnecessary cost. Our findings of over 20% of patients receiving laxatives and/or enemas/suppositories before diarrhoeal episodes suggest a need for rational prescribing during diarrhoeal episodes to reduce unnecessary burden on critically ill patients.

## Conclusions

Diarrhoea was common on our ICU with a prevalence of 12.9%. Adjusting for baseline differences, patients suffering diarrhoea experienced increased ICU length of stay and increased ICU mortality compared with patients without diarrhoea.

A low yield of stool investigations and low prevalence of *Clostridium difficile* and norovirus suggest a possible pathogenic role for non-infective processes. Several patients received laxatives and enemas before diarrhoeal episodes suggesting a need for rational intensivist prescribing.

Further studies are warranted to establish the pathogenesis of gastrointestinal dysfunction in critically ill patients, in order to develop evidence-based management plans for reducing the incidence of diarrhoea, and its clinical and financial impact.

## Methods

### Study design and setting

University College Hospital (UCH) is an 846-bed Central London teaching hospital with over 120,000 hospital admissions per year. Departmental computerised patient records were analysed to identify all patients aged >18 years admitted for level 2 or 3 care to its 35-bed mixed medical/surgical adult ICU, in the 60 months between 01/01/2006 and 31/12/2010. During this time, UCH provided surgery for all major specialties except cardiothoracic and neurosurgery. In 2009, UCH opened a Post-Anaesthesia Care Unit as part of its critical care facilities, which saw an increase in post-surgical ICU admissions.

Our study was reviewed by the University College London Joint Research Office who deemed this study a service evaluation which did not require formal ethics approval. Consent was not required as our study involved retrospective analysis of anonymous, routinely collected group data from University College Hospital.

### Data collection

A procedure for sending stool samples is clearly defined for UCH ICU, with samples being sent for microbiological/virological assessment in the case of loose stools, according to the Bristol Stool Chart^27^. Patients with such samples sent during their ICU admission were deemed to have suffered diarrhoea.

The index stool samples for each patient admission were analysed for stool microscopy and culture, *Clostridium difficile* toxin A and B using an immunoassay enzyme^28^, *Clostridium difficile* glutamate dehydrogenase antigen, and virology. The choice of gastrointestinal virus selected for analysis was guided by clinical history (e.g. vomiting and diarrhoea, community or outbreak acquisition). A standard screening panel (since 2000) includes Norovirus 1 and 2, Rotavirus and Adenovirus. *Clostridium difficile* antigen testing was introduced during the study period at UCH in June 2009, and added to our *Clostridium difficile* toxin testing. Presence of *Clostridium difficile* antigen indicates carriage of a potentially toxin-producing organism which may develop into disease. Presence of *Clostridium difficile* toxin is most likely to be associated with disease. Both *Clostridium difficile* toxin-positive and toxin-negative/antigen-positive samples were classified as positive infectious samples. Stool sample results were compiled and analysed with data collected from computerised patient records.

Demographic data, including age, sex, admissions category (medical or surgical), Acute Physiology and Chronic Health Evaluation (APACHE) II score^29^ and reason for ICU admission, were collected. Laxatives (Lactulose, Senna, Polyethylene Glycol, Docusate Sodium, Ispaghula Husk, Sodium Picosulfate with Magnesium Citrate), suppositories (Glycerol) and enemas (Phosphates) received during patient ICU admission were also recorded. Outcome variables recorded for all patient admissions were ICU LOS and ICU mortality.

### Statistical analysis

Patient data were extracted from the hospital database, compiled with stool sample results, and analysed using Microsoft Access 2010 (Microsoft, Redmond, Washington, USA) and Stata Version 13 (StataCorp, Texas, USA). Data were compared between patients suffering diarrhoea during their admission and patients not suffering diarrhoea during their admission. Continuous variables were analysed using the Mann-Whitney U test or Student’s t-test and categorical data were analysed using Fisher’s exact test. Multivariate models were used to adjust for baseline characteristics (age, admission category, APACHE II, operative intervention and other variables that were significantly different between the two groups) when examining the effect of diarrhoea on ICU LOS and mortality. For ICU LOS a multiple regression model was used to estimate the difference in LOS. As LOS was not normally-distributed, we also used an ordinal logistic regression model to obtain an odds ratio for an increased LOS. Cox proportional hazard models were used to assess the relationship between ICU LOS from the time of diarrhoea diagnosis, and to assess mortality risk during ICU admission in admissions with and without diarrhoea. Diarrhoea was fitted as a time-dependent variable in these models in order that attribution of increased LOS to the presence of diarrhoea could be discerned from increased LOS as a risk factor for developing diarrhoea. As the proportional hazards assumption was violated for time to discharge, data was additionally split at each failure time and the analysis was stratified by time. A P-value of less than 0.05 was considered statistically significant.

## Additional Information

**How to cite this article**: Tirlapur, N. *et al*. Diarrhoea in the critically ill is common, associated with poor outcome, and rarely due to *Clostridium difficile. Sci. Rep.* **6**, 24691; doi: 10.1038/srep24691 (2016).

## Supplementary Material

Supplementary Information

## Figures and Tables

**Table 1:** Demographic and baseline characteristics for diarrhoea and non-diarrhoea groups.

	**Admissions with Diarrhoea**^a^ **(N = 1207)**	**Admissions with No Diarrhoea**^a^ **(N = 8124)**	***P*****-value**
**Age** [mean (standard deviation)]	60.8 (16.5)	58.3 (17.8)	<0.001
**Male sex**	678 (56.2%)	4457 (54.9%)	0.40
**Referring Specialty**			
Medical	726 (60.1%)	2358 (29.0%)	<0.001
Surgical	481 (39.9%)	5766 (71.0%)	
**APACHE II score**^b^ [median (interquartile range)]	22 (17–28)	16 (11–21)	<0.001
**Reason for Intensive Care Unit admission**			
Cardiovascular	109 (9.0%)	487 (6.0%)	<0.001
Respiratory failure	351 (29.1%)	878 (10.8%)	<0.001
Haemorrhage	81 (6.7%)	556 (6.8%)	0.85
Sepsis	200 (16.6%)	455 (5.6%)	<0.001
Renal failure	67 (5.6%)	138 (1.7%)	<0.001
Operative intervention	228 (18.9%)	4735 (58.3%)	<0.001
Neurological	91 (7.5%)	534 (6.6%)	0.20
Haematological	40 (3.3%)	99 (1.2%)	<0.001
Liver failure	17 (1.4%)	60 (0.7%)	0.03
Other	23 (1.9%)	182 (2.2%)	0.53

^a^Data shown as N (%) unless stated otherwise.

^b^Acute Physiology and Chronic Health Evaluation (APACHE) II score.

**Table 2:** Admissions of patients suffering diarrhoea comparing admissions with stool samples positive vs negative for *Clostridium difficile*.

**Admissions Suffering Diarrhoea During Their ICU Stay (N = 1207)**	**Number (%)**	**ICU Mortality (%)**
*Clostridium difficile* positive	97 (8.0)	26 (26.8)
Glutamate dehydrogenase antigen positive only	27 (2.2)	3 (11.1)
Toxin positive	70 (5.8)	23 (32.9)
*Clostridium difficile* negative	1112 (92.1)	240 (21.6)
Preceding laxative and/or enema use	305 (25.3)	71 (23.3)
Negative stool sample	793 (65.7)	169 (21.3)
Positive stool sample	14 (1.2)	0 (0)

**Table 3:** Ordinal logistic regression analysis for length of intensive care unit stay for admissions with vs without diarrhoea.

**Co-variates**^a^	**Odds Ratio (95% Confidence Interval) for Increased Intensive Care Unit Stay**	***P-*****value**
**Age** (per 10 year increase)	0.99 (0.96–1.01)	0.20
**Surgical: Medical**	1.08 (0.95–1.21)	0.23
**Quintile of APACHE II**^b^		
1	1.00	<0.001
2	1.95 (1.74–2.19)	
3	2.87 (2.55–3.23)	
4	4.30 (3.81–4.86)	
5	6.56 (5.74–7.49)	
**Reason for Intensive Care Unit admission**		
Operative intervention	1.00	
Cardiovascular	1.81 (1.51–2.17)	<0.001
Respiratory failure	3.27 (2.83–3.78)	<0.001
Haemorrhage	1.13 (0.96–1.32)	0.14
Sepsis	2.28 (1.92–2.70)	<0.001
Renal failure	1.93 (1.48–2.52)	<0.001
Neurological	1.34 (1.11–1.61)	0.002
Haematological	1.66 (1.19–2.31)	0.003
Liver failure	3.73 (2.50–5.58)	<0.001
Other	1.66 (1.29–2.14)	<0.001
**Diarrhoea vs. Non-Diarrhoea**	9.48 (8.32–10.81)	<0.001

^a^Ordinal logistic regression analysis: dependent variable is length of ICU stay in days.

^b^Acute Physiology and Chronic Health Evaluation II score.

**Table 4:** Analysis for intensive care unit (ICU) mortality for admissions of patients suffering vs not suffering diarrhoea during their ICU stay.

**Co-variates**^a^	**Odds Ratio (95% CI) for mortality**	***P-*****value**
**Age** (per 10 year increase)	1.18 (1.13–1.23)	<0.001
**Surgical: Medical**	0.52 (0.43–0.62)	<0.001
**Quintile of APACHE-II**^b^		
1	1.00	
2	0.32 (0.23–0.46)	
3	0.46 (0.35–0.61)	<0.001
4	0.72 (0.58–0.90)	
5	1.27 (1.04–1.56)	
**Reason for ICU admission**		
Operative intervention	1.00	
Cardiovascular	2.99 (2.26–3.95)	<0.001
Respiratory failure	2.01 (1.55–2.59)	<0.001
Haemorrhage	1.77 (1.28–1.44)	<0.001
Sepsis	1.75 (1.31–2.34)	<0.001
Renal failure	1.49 (1.02–2.20)	0.04
Neurological	1.96 (1.43–2.69)	<0.001
Haematological	1.98 (1.23–3.21)	0.005
Liver failure	3.23 (1.91–5.47)	<0.001
Other	2.12 (1.32–3.42)	0.002
**Diarrhoea vs. Non-Diarrhoea**	1.99 (1.70–2.32)	<0.001

^a^Cox proportional hazard model with diarrhoea as a time dependent co-variate.

^b^Acute Physiology and Chronic Health Evaluation II score.
