# Supplementary material for: Diarrhoea in the critically ill is common, associated with poor outcome, and rarely due to Clostridium difficile
Source: Sci Rep. 2016 Apr 20;6:24691. doi: 10.1038/srep24691 (PMC4837391; doi:10.1038/srep24691)

Diarrhoea in the critically ill is common, associated with poor outcome, and rarely due to *Clostridium difficile*

Nikhil Tirlapur[1*], Zudin A. Puthucheary[2,3,4], Jackie A. Cooper[5], Julie Sanders[6], Pietro G. Coen[7], S. Ramani Moonesinghe[8], A. Peter Wilson[9], Michael G. Mythen[10], Hugh E. Montgomery[3,11]

[1]Section of Anaesthetics, Pain Medicine & Intensive Care, Faculty of Medicine, Imperial College London, 369 Fulham Road, London, SW10 9NH, UK

[2]Critical Care, University College London Hospitals NHS Foundation Trust, London, UK

[3]Institute for Sport Exercise and Health, University College London, London, UK

[4]Respiratory and Critical Care Medicine, University Medicine Cluster, National University Health System, Singapore

[5]Centre for Cardiovascular Genetics, Institute of Cardiovascular Science, University College London, London, UK

[6]St Bartholomew's Hospital, Barts Health NHS Trust, London, UK

[7]Infection Control Office, University College London Hospitals NHS Foundation Trust, London, UK

[8]University College London Hospitals NHS Foundation Trust / University College London National Institute for Health Research Surgical Outcomes Research Centre, London, UK

[9]Microbiology & Virology, University College London Hospitals NHS Foundation Trust, London, UK

[10]University College London Hospitals NHS Foundation Trust / University College London National Institute for Health Research Biomedical Research Centre, London, UK

[11]Critical Care, Whittington Hospital, University College London, London, UK

Supplementary Table S1. Relationship between type of surgery (gastrointestinal vs non-gastrointestinal) and diarrhoea.

	**Admissions with Diarrhoea**[a] (N = 1207)	**Admissions with No Diarrhoea**[a] (N = 8124)	***P*-value**
**Referring Specialty**			
Medical	726 (60.1%)	2358 (29.0%)	
Surgical	481 (39.9%)	5766 (71.0%)	
GI surgery	212 (17.6%)	2533 (31.2%)	0.96
Non-GI surgery	269 (22.3%)	3233 (39.8%)	
**Reason for Intensive Care Unit admission**			
Operative intervention	228 (18.9%)	4735 (58.3%)	
GI surgery	107 (8.9%)	2155 (26.5%)	0.68
Non-GI surgery	121 (10.0%)	2580 (31.8%)	

[a]Data shown as N (%) unless stated otherwise.

Supplementary Table S2. Reasons for intensive care unit admission in patients admitted following gastrointestinal surgery and suffering diarrhoea.

	**GI Surgery Admissions with Diarrhoea**[a] (N = 212)
**Reason for ICU Admission**	
Operative intervention	102 (48.1%)
Cardiovascular	13 (6.1%)
Respiratory failure	25 (11.8%)
Haemorrhage	16 (7.5%)
Sepsis	49 (23.1%)
Renal failure	1 (0.5%)
Neurological	1 (0.5%)
Haematological	0 (0.0%)
Liver failure	2 (0.9%)
Other	3 (1.4%)

[a]Data shown as N (%) unless stated otherwise.

Supplementary Table S3. Reasons for operative intervention in patients suffering diarrhoea following admission to the intensive care unit due to operative intervention after gastrointestinal surgery.

	**GI Surgery Admissions with Diarrhoea[a]** (N = 102)
**Reason for Operative Intervention**	
Emergency	55 (53.9%)
Elective	47 (46.1%)
Tumour	27 (26.5%)
Fistula	10 (9.8%)
Congenital malformation	4 (3.9%)
Hernia	2 (2.0%)
Bariatric	2 (2.0%)
Inflammatory bowel disease	2 (2.0%)

[a]Data shown as N (%) unless stated otherwise.

Supplementary Table S4. Study population results per year from 2006 to 2010.

	**2006** (N=1551)	**2007** (N=1620)	**2008** (N=1837)	**2009** (N=2098)	**2010** (N=2225)
**Admissions Suffering Diarrhoea During ICU Stay**	214	262	253	238	240
**Prevalence of Diarrhoea**	**13.8%**	**16.2%**	**13.8%**	**11.3%**	**10.8%**
**Admissions with *C Difficile***	23	16	7	19	32
**Prevalence of *C Difficile***	**1.5%**	**1.0%**	**0.4%**	**0.9%**	**1.4%**
**Mean Age** Years [SD]	57.8 [17.9]	58.6 [17.9]	58.5 [17.7]	58.6 [17.8]	59.3 [17.3]
**Male Sex**	917 (59.1%)	935 (57.7%)	994 (54.1%)	1088 (51.9%)	1201 (54.0%)
**Referring Specialty**					
Medical	614 (39.6%)	625 (38.6%)	653 (35.5%)	626 (29.8%)	566 (25.4%)
Surgical	937 (60.4%)	995 (61.4%)	1184 (64.5%)	1427 (70.2%)	1659 (74.6%)
**Median APACHE II** [IQR]	18 [13-23]	17 [12-23]	16 [12-22]	17 [12-22]	14 [7-20]
**Cause for ICU Admission** (no [%])					
Cardiovascular	106 (6.8%)	121 (7.5%)	117 (6.4%)	131 (6.2%)	121 (5.4%)
Respiratory Failure	219 (14.1%)	223 (13.8%)	294 (16.0%)	263 (12.5%)	230 (10.3%)
Haemorrhage	132 (8.5%)	129 (8.0%)	120 (6.5%)	130 (6.2%)	126 (5.7%)
Sepsis	151 (9.7%)	135 (8.3%)	128 (7.0%)	106 (5.1%)	135 (6.1%)
Renal Failure	41 (2.6%)	37 (2.3%)	52 (2.8%)	44 (2.1%)	31 (1.4%)
Operative intervention	667 (43.0%)	778 (48.0%)	935 (50.9%)	1200 (57.2%)	1383 (62.2%)
GI Surgery	*233 (15.0%)*	*361 (22.3%)*	*509 (27.7%)*	*597 (28.5%)*	*562 (25.3%)*
Non-GI Surgery	*434 (28.0%)*	*417 (25.7%)*	*426 (23.2%)*	*603 (28.7%)*	*821 (36.9%)*
Neurological	149 (9.6%)	118 (7.3%)	92 (5.0%)	138 (6.6%)	128 (5.8%)
Haematological	23 (1.5%)	21 (1.3%)	35 (1.9%)	29 (1.4%)	31 (1.4%)
Liver Failure	16 (1.0%)	11 (0.7%)	18 (1.0%)	20 (1.0%)	12 (0.5%)
Other	47 (3.0%)	47 (2.9%)	46 (2.5%)	37 (1.8%)	28 (1.3%)
**Median ICU LOS** Days [IQR]	1.8 [0.9-4.9]	2.2 [1.0-5.4]	2.0 [1.0-4.8]	1.8 [0.9-4.0]	1.8 [1.0-3.9]
**ICU Mortality** (no [%])	224 (14.4%)	210 (13.0%)	175 (9.5%)	188 (9.0%)	173 (7.8%)

Supplementary Figure I. Prevalence of diarrhoea over study period unadjusted vs adjusted for reason for ICU admission and APACHE-II score.

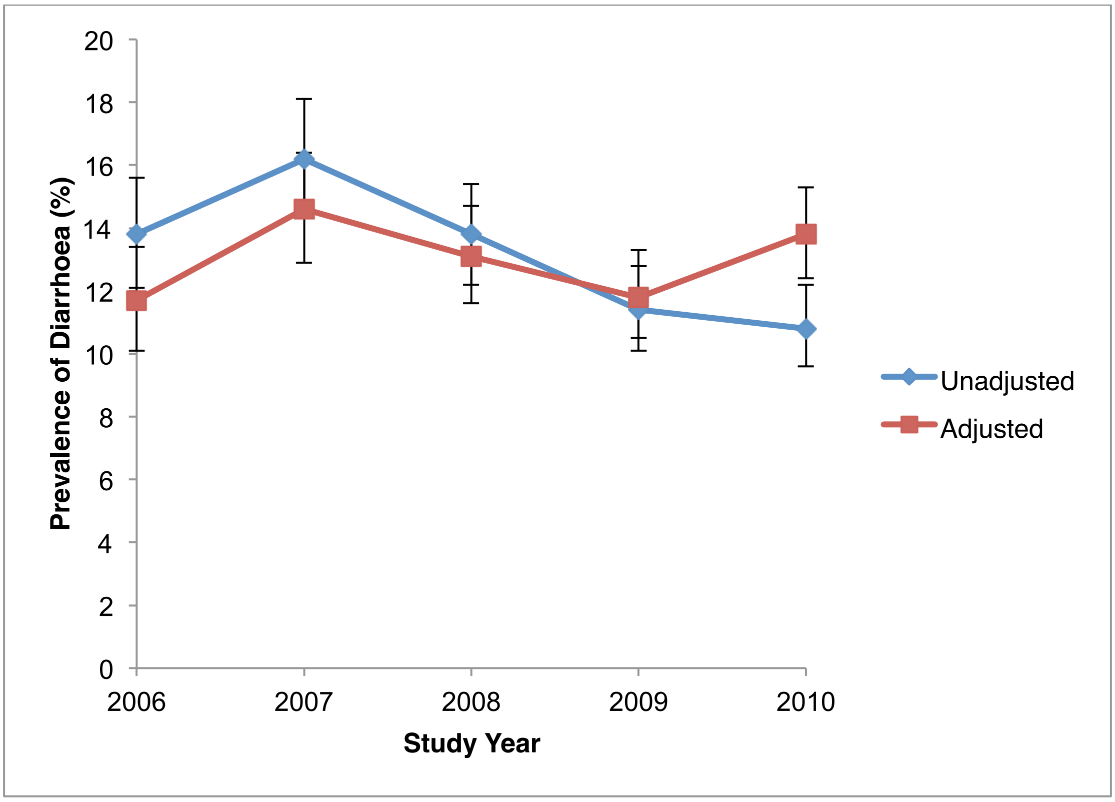

Supplement: Supplementary Information [file srep24691-s1.pdf]
